# Long-Range Enhancer Interactions Are Prevalent in Mouse Embryonic Stem Cells and Are Reorganized upon Pluripotent State Transition

**DOI:** 10.1016/j.celrep.2018.02.040

**Published:** 2018-03-06

**Authors:** Clara Lopes Novo, Biola-Maria Javierre, Jonathan Cairns, Anne Segonds-Pichon, Steven W. Wingett, Paula Freire-Pritchett, Mayra Furlan-Magaril, Stefan Schoenfelder, Peter Fraser, Peter J. Rugg-Gunn

**Affiliations:** 1 Epigenetics Programme, Babraham Institute, Cambridge CB22 3AT, UK; 2 Nuclear Dynamics Programme, Babraham Institute, Cambridge CB22 3AT, UK; 3 Bioinformatics Group, Babraham Institute, Cambridge CB22 3AT, UK; 4 Department of Biological Science, Florida State University, Tallahassee, FL 32306, USA; 5 Wellcome Trust – Medical Research Council Cambridge Stem Cell Institute, University of Cambridge, Cambridge CB2 1QR, UK

**Keywords:** epigenetics, genome organization, chromatin looping, gene regulation, pluripotency, differentiation, promoter capture Hi-C

## Abstract

Transcriptional enhancers, including super-enhancers (SEs), form physical interactions with promoters to regulate cell-type-specific gene expression. SEs are characterized by high transcription factor occupancy and large domains of active chromatin, and they are commonly assigned to target promoters using computational predictions. How promoter-SE interactions change upon cell state transitions, and whether transcription factors maintain SE interactions, have not been reported. Here, we used promoter-capture Hi-C to identify promoters that interact with SEs in mouse embryonic stem cells (ESCs). We found that SEs form complex, spatial networks in which individual SEs contact multiple promoters, and a rewiring of promoter-SE interactions occurs between pluripotent states. We also show that long-range promoter-SE interactions are more prevalent in ESCs than in epiblast stem cells (EpiSCs) or *Nanog*-deficient ESCs. We conclude that SEs form cell-type-specific interaction networks that are partly dependent on core transcription factors, thereby providing insights into the gene regulatory organization of pluripotent cells.

## Highlights

• Promoter-capture Hi-C identifies 3D interactions in mouse pluripotent cells
• Super-enhancers (SEs) form complex spatial networks contacting multiple promoters
• Rewiring of promoter-SE interactions between ESC and EpiSC pluripotent states
• Long-range SE interactions are a hallmark of mouse ESCs

## Graphical Abstract

**Figure:**
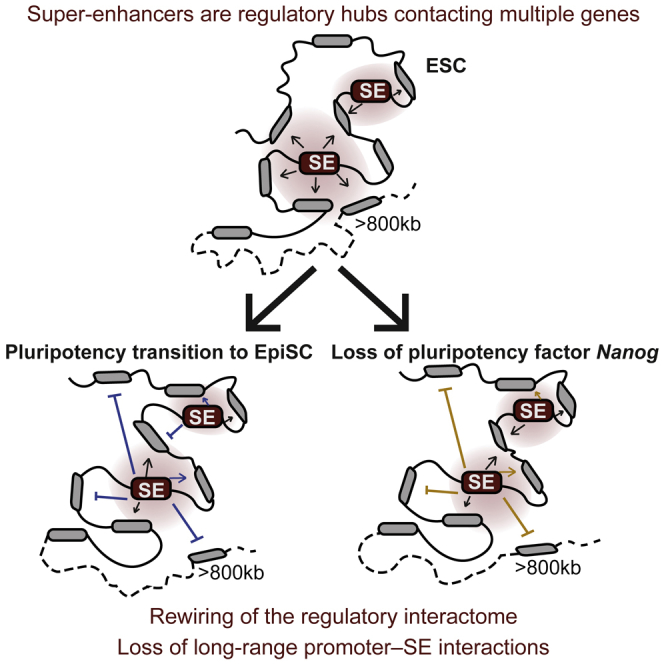

## Teaser

Novo et al. use promoter-capture Hi-C to map the target promoters of super-enhancers (SEs) in mouse pluripotent cells. SEs form complex networks, and a subset of promoter-SE interactions was rewired between ESCs and EpiSCs. In ESCs, many SEs form long-range contacts that are not detected in EpiSC or *Nanog*-deficient ESCs.

## Introduction

Complex, multi-layered compaction and folding enables the eukaryotic genome to undergo functional organization within the 3D nuclear space. Higher-order chromatin architecture forms into topologically associating domains (TADs), which are discrete ∼1-Mb structures that compartmentalize and insulate the genome (Nora et al., 2012, Dixon et al., 2012). Within TADs, DNA loops bring promoters and their distal regulatory elements into close physical proximity. TAD organization is orchestrated by architectural proteins like CTCF and cohesin and is largely cell-type invariant (Rao et al., 2014, Wei et al., 2013, Apostolou et al., 2013, Nora et al., 2017). In contrast, intra-TAD DNA loops are typically cell-type specific and are frequently rewired upon cell state changes (Smith et al., 2016, Denholtz et al., 2013, Schoenfelder et al., 2015b, Freire-Pritchett et al., 2017). This hierarchical nuclear organization permits the coordinated activation and repression of cell-identity genes while restricting the pool of promoters that are able to contact regulatory elements, including transcriptional enhancers (Dixon et al., 2012).

The mechanisms responsible for establishing the interactions between promoters and their regulatory elements include the binding of cell-type-specific transcription factors and the local chromatin landscape (reviewed in Heinz et al., 2015, Vernimmen and Bickmore, 2015). In mouse embryonic stem cells (ESCs), global, low-resolution analyses showed that large regions harboring clusters of NANOG, OCT4, or SOX2 binding sites preferentially interact, and depleting NANOG or OCT4 reduces the frequency of interactions (de Wit et al., 2013, Denholtz et al., 2013). In addition, the recruitment of NANOG to an ectopic site is sufficient to bring different distant regions together, thereby demonstrating a direct role for pluripotency factors in controlling chromatin topology (de Wit et al., 2013). At higher resolution, pluripotent-specific interactomes around the *Nanog* and *Pou5f1* promoters have been mapped (Apostolou et al., 2013), and the maintenance of the chromatin structure at these sites depends on pluripotency factors such as OCT4 (Levasseur et al., 2008). Contacts are also sensitive to differentiation cues that can disrupt promoter-enhancer loops at these loci (Kagey et al., 2010, Gaspar-Maia et al., 2011, Phillips-Cremins et al., 2013). In addition, transcription-factor-induced reprogramming to pluripotency induces a reorganization in the chromatin topology of the donor somatic cells (Krijger et al., 2016, Beagan et al., 2016). These studies collectively provide support for a model where DNA looping at regulatory elements can be driven and/or regulated by pluripotency-associated transcription factors.

A subset of enhancers, known as super-enhancers (SEs; Hnisz et al., 2013, Whyte et al., 2013) or stretch enhancers (Parker et al., 2013), was proposed to be crucial for regulating the expression of cell identity genes. SEs form large domains (typically >3 kb) with high levels of the active enhancer mark, histone 3 lysine 27 acetylation (H3K27ac), and are densely occupied by key transcription factors (Whyte et al., 2013). In ESCs, for example, most SEs are bound by NANOG, OCT4, and SOX2 (Whyte et al., 2013). SEs are thought to control gene expression programs by associating with promoters and modulating their transcriptional output. The gene promoter targets of SEs are typically predicted using algorithms that account for linear proximity and high levels of gene expression (Whyte et al., 2013). This approach in ESCs assigned 231 SEs to 210 genes; the majority of these genes have prominent roles in controlling the pluripotent state. Thus, the binding of cell-specific transcription factors to SEs is predicted to establish self-regulatory feedback that may stabilize cell identity.

The function of several SEs has been examined in ESCs using genetic approaches. Deleting multiple SEs in the vicinity of the *Nanog* and *Sox2* loci leads to variable effects on predicted target gene transcription (Zhou et al., 2014, Blinka et al., 2016). This phenotypic variability following SE deletion was also observed in another study, which reported a large range in the transcriptional misregulation of predicted target genes (Moorthy et al., 2017). Interestingly, deleting a SE can also alter the transcription of genes that are not currently assigned to that SE, suggesting that SEs might provide regulatory inputs to multiple promoters and operate within larger networks or interaction hubs (Blinka et al., 2016, Moorthy et al., 2017). The underlying causes of the observed transcriptional variability are not clear but may be due to the partial redundancy of the individual enhancer elements that comprise a SE and to the failure of current SE predictions to infer additional strong regulatory associations. Mapping the precise regulatory inputs to key cell identity genes will improve the curation of transcriptional networks and lead to a better understanding of the phenotypic variability that is observed upon SE perturbations.

Despite the progress in defining regulatory elements within the genome, inferring promoter-SE associations based on linear proximity has several shortcomings. First, regulatory elements are frequently located at considerable distances away from their target promoters in linear DNA (reviewed in van Heyningen and Bickmore, 2013). Second, promoters are often contacted by multiple regulatory elements, and individual regulatory elements may interact with multiple target promoters, either sequentially or simultaneously (Sanyal et al., 2012, Freire-Pritchett et al., 2017, Schoenfelder et al., 2015a). This complexity can easily be overlooked when assigning single promoter-SE pairings. However, methods such as chromosome conformation capture (3C) approaches map the physical interactions between promoters and their regulatory elements, including SEs. Promoter-capture Hi-C (PCHi-C) in ESCs identified interactions between SEs and 503 genes (Schoenfelder et al., 2015a), which is many more than the predicted number of 210 genes, thereby pointing toward a more complex spatial network than previously thought. It is important to expand these results by carefully examining this spatial network in more detail, including an assessment of the combinatorial promoter-SE interactions, and in additional cell types. Moreover, it remains unknown how promoter-SE interactions change upon developmental state transitions, and how transcription factors may establish or maintain promoter-SE interactions. These points are important to address in order to assess the accuracy of current SE target gene assignments, understand the complexities of regulatory networks that underpin cell identify, and better interpret current and future functional experiments.

ESCs require precise regulation of transcriptional programs to enable the balance between self-renewal and effective differentiation and provide an informative system to study how gene regulatory interactions are altered on cell state change. Here, we used PCHi-C to identify promoter-based interactions in ESCs, and we focused our analysis on investigating the interactions with SEs. We assigned promoter targets to previously defined SEs, which both confirmed and extended the number of known regulatory contacts. We also mapped promoter-SE interactions in a more developmentally advanced pluripotent cell type, epiblast stem cells (EpiSCs), and found that a subset of interactions was rewired between ESCs and EpiSCs. Interestingly, promoter-SE interactions frequently spanned large distances (>800 kb) in ESCs, but not in EpiSCs or in *Nanog*-deficient ESCs. Together, these results provide insights into the organization of chromatin topology in ESCs and lead to a better understanding of how gene regulatory networks can switch between pluripotent states.

## Results

### SEs Are Highly Interactive Hubs in ESCs

To map the gene promoters that are in close physical proximity to SEs in mouse ESCs, we performed PCHi-C, a high-throughput 3C-based technique with a capture step to enrich for interactions at >22,000 promoters at single-restriction-fragment resolution (Mifsud et al., 2015, Schoenfelder et al., 2015a). We identified significant interactions using Capture Hi-C Analysis of Genomic Organization (CHiCAGO) (Cairns et al., 2016), including several previously reported contacts between promoters and their regulatory elements in ESCs for *Pax2*, *Tbx5*, and *Wnt6* (Figure S1; Schoenfelder et al., 2015a, Schoenfelder et al., 2015b).

**Figure 1:**
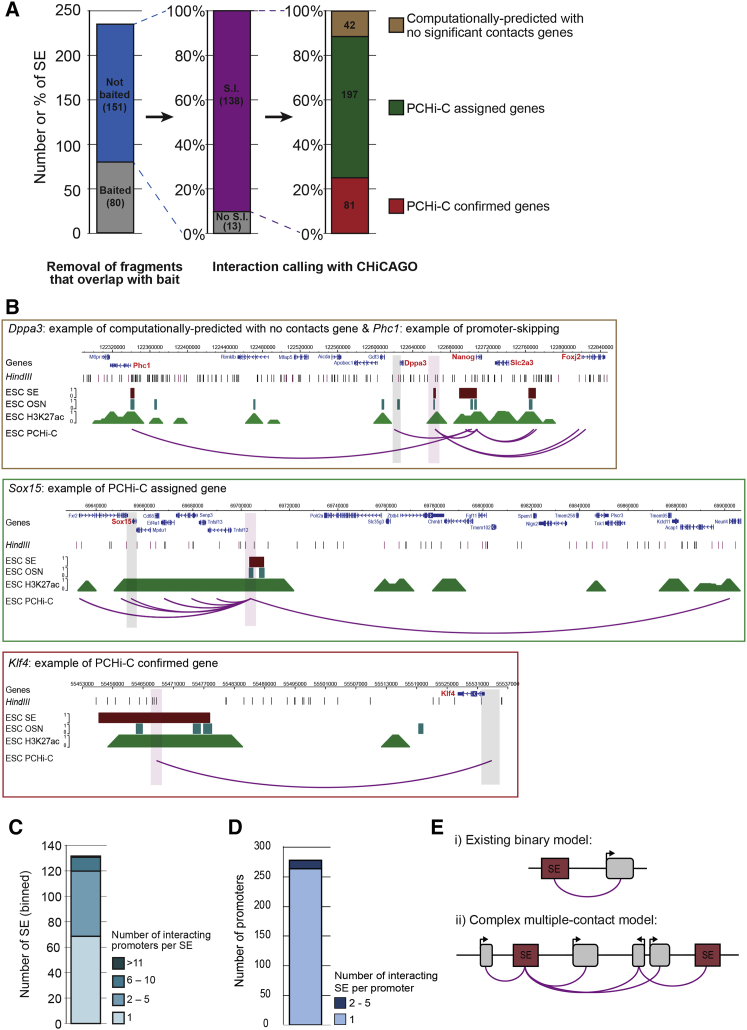
Promoter-Capture Hi-C Reveals that SEs Form Spatial Networks in Mouse ESCs (A) From the 231 previously defined SEs in ESCs (Whyte et al., 2013), 80 overlapped baited Hi-C fragments and were not considered further in this analysis. Significant promoter-SE interactions were identified at 138 of the remaining SEs, and a comparison of SE target genes between PCHi-C and computational assignments is shown. (B) Example of a computationally predicted SE-target gene (*Dppa3*) without significant PCHi-C interactions (top). Examples of a newly identified SE-target gene (*Sox15*; middle) and a computationally predicted SE-target gene that was confirmed by PCHi-C (*Klf4*; bottom). Tracks show HindIII sites (black lines), baited HindIII sites (purple lines), ESC SEs (maroon), and ESC OSN (OCT4, SOX2, and NANOG) binding sites (teal). Arcs show significant interactions. Red and gray vertical bars highlight the location of SEs and promoters, respectively. The upper genome browser screenshot also exemplifies SE promoter skipping, where the SE close to *Phc1* contacts the *Nanog* promoter, skipping over six genes. (C) Stacked bar chart showing the number of SEs interacting with 1, 2–5, 6–10, or >11 gene promoters. (D) Stacked bar chart showing the number of gene promoters interacting with 1 or 2–5 SEs. (E) Schematic proposing an updated model of SE interactions from a binary promoter-SE interaction (i) to a more complex network where SEs can contact multiple promoters, and promoters can interact with more than one SE (ii). Red and gray rectangles represent SEs and genes, respectively. See also Figure S1 and Table S1.

We focused on the 901 HindIII fragments overlapping the 231 SEs that were defined previously in ESCs (Whyte et al., 2013). We assigned each SE to one or more gene promoters based on their interactions (Table S1). We restricted our analysis to the 151 SEs (∼70%) that did not overlap with a promoter to ensure that detected interactions were mediated by promoter-SE, and not by promoter-promoter, contacts (Figure 1A). Of these, we detected significant promoter interactions with 138 SEs (91%). The original description of SEs in ESCs assigned 210 genes to SEs by linear proximity (Whyte et al., 2013). Our PCHi-C data provide direct evidence for promoter-SE interactions at 81 of those genes, including pluripotency-associated factors such as *Klf4, Sox2, Nanog*, and *Fgf4* (Figures 1A and 1B; Table S1). No significant SE contacts were detected for 42 genes assigned to a SE by computational predictions, confirming that linear distance does not accurately infer regulatory contacts (Figures 1A and 1B; Table S1). Excitingly, we identified an additional 197 target promoters that have not been previously associated with a SE in ESCs (Figures 1A and 1B; Table S1). Notably, several newly identified genes encode for components of the glycolysis pathway, which is highly active in serum-grown mouse ESCs (Zhang et al., 2012, Zhang et al., 2016). Mapping the SE-interactome in ESCs, therefore, has provided direct evidence for predicted promoter-SE contacts, and expanded the gene regulatory networks to reveal an unanticipated connection with the control of metabolic activity.

Further analysis of the PCHi-C data revealed frequent promoter skipping by SEs, as exemplified by the SE proximal to *Phc1* that contacts the more distal *Nanog* promoter, skipping over six other promoters (Figure 1B, upper). This observation is similar to the skipping of active promoters by “regular” enhancers (Schoenfelder et al., 2015a). We also observed that half of the interacting SEs contacted two or more promoters, with ∼5% interacting with >6 different promoters (Figure 1C). Most promoters contacted one individual SE, and only 14 promoters contacted 2–5 SEs (Figure 1D). Together, this high-resolution contact map shows that SEs are highly interactive and complex hubs in ESCs, where several promoters can be in close physical contact to an individual SE (Figure 1E).

### Rewiring of the SE Interactome upon Pluripotent State Transition

**Figure 2:**
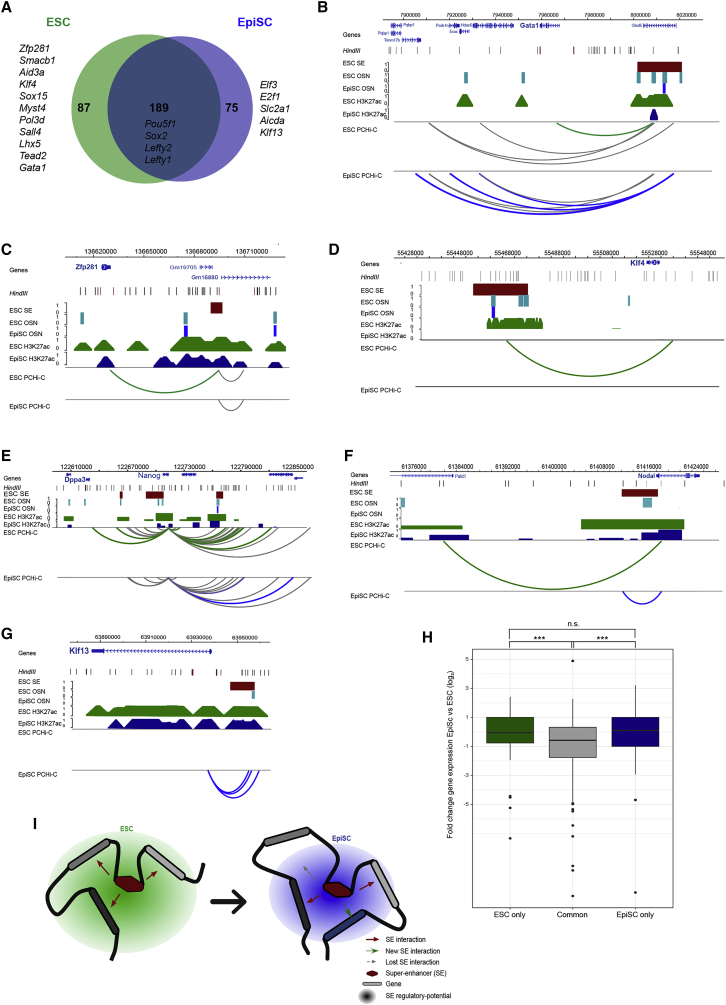
Pluripotent State-Specific Wiring of SE Interactions (A) The promoters in close physical proximity to ESC SEs were largely maintained in EpiSCs, but subsets of significant SE interactions were detected uniquely in each cell type. (B–G) Differences in the SE interactome between ESCs and EpiSCs are shown for the *Gata1* (B), *Zfp281* (C), *Klf4* (D), *Nanog* (E), *Nodal* (F), and *Klf13* (G) loci. Arcs indicate significant interactions colored by cell-type specificity (ESC only, green; EpiSC only, blue; shared, gray). (H) Boxplot showing the log2 fold change in gene expression between EpiSCs and ESCs, for genes interacting with SEs only in ESCs, in ESCs and EpiSCs, and only in EpiSCs. ^∗∗^p < 0.01 (Mann-Whitney *U* test). (I) Model illustrating the rewiring of interactions between pluripotent states at an individual SE. The red arrows represent the physical contacts between promoters detected by PCHi-C, creating a spatial hub for potential SE regulation (green/blue circles). Upon transitioning to EpiSCs, some contacts are lost (gray arrow), while new ones are established (green arrow). See also Figure S2 and Table S1.

ESCs and EpiSCs represent functionally distinct pluripotent states with differing transcriptional and epigenetic programs that reflect their similarities to pre- and post-implantation epiblast cells, respectively. Many enhancers undergo changes in activity between the two pluripotent cell types (Buecker et al., 2014, Factor et al., 2014), and transcriptional differences include the reduced expression of genes such as *Nanog* and *Klf4* (Brons et al., 2007, Osorno et al., 2012, Tesar et al., 2007, Guo et al., 2009, Novo et al., 2016). To investigate how the ESC SE interactome is reorganized upon this developmental transition, we performed PCHi-C in EpiSCs. We found that the majority (70%) of genes interacting with SEs in ESCs were the same in EpiSCs (Figure 2A). These genes encode key pluripotency factors such as *Pou5f1* and *Sox2*, plus signaling regulators like *Lefty1* and *Smad1* (Figure 2A).

The remaining 30% of SE-interacting genes were specific to each pluripotent state (Figure 2A). There were 87 genes that significantly interacted with SEs only in ESCs, including *Sall4*, *Tead2*, *Zfp281*, *Klf4*, *Sox15*, and *Gata1* (Figures 2A–2E; Table S1). We also detected in EpiSCs a set of promoters that gained significant interactions with ESC SEs, including *Aicda* and *Klf13* (Figures 2A, 2F, and 2G; Table S1). The majority (92%) of these SEs remained either a SE or an active enhancer in EpiSCs. Interestingly, two-thirds of the EpiSC-specific gene promoters are interacting with a SE that is in contact with a different gene promoter in ESCs. This suggests that the same SE can interact with different promoters in different cell types. Although there was no global association between cell-type-specific SE interaction and target gene expression (Figure 2H), the rewiring of a set of ESC SE interactions was consistent with transcriptional changes at individual loci. For example, loss of contacts between the *Klf4* promoter and a SE, and between the *Dppa3* promoter and a SE, is consistent with their transcriptional downregulation in EpiSCs compared to ESCs (Figures 2D and 2E). In addition, the gain of a significant interaction in EpiSCs between the *Nodal* promoter and its pluripotent-specific enhancer (Papanayotou et al., 2014) is in line with the transcriptional upregulation of *Nodal* in EpiSCs (Figure 2F). Taken together, these results show that the majority of promoter-SE interactions are conserved between ESCs and EpiSCs, but a subset of the ESC SE interactome is reorganized upon the transition to an EpiSC state (Figure 2I).

We examined ESC chromatin immunoprecipitation sequencing (ChIP-seq) data (Sánchez-Castillo et al., 2015) to identify proteins that were enriched at the gene promoters in contact with SEs only in ESCs (ESC only). This revealed that ESC-only promoters are commonly bound by pluripotency factors, including TFCP2L1, KLF4, PRDM14, and SOX2 (Figure S2A). We also examined the gene promoters that are not in contact with SEs in ESCs but acquire SE interactions in EpiSCs (EpiSC only). This category of promoters was enriched for a different set of proteins that included JARID2, EZH2, TBX3, TET1, and KDM2A (Figure S2A). Core factors, such as OCT4 and MYC, were present at similar levels for ESC-only and EpiSC-only gene promoters. The analysis indicates that different categories of SE-interacting promoters are associated with distinct sets of transcription factor occupancy. This configuration might help to stabilize active SE interactions in ESCs and also promote the “priming” of new SE interactions upon transition to EpiSCs.

### Long-Range SE Interactions Are Detected in ESCs, but Not in EpiSCs

**Figure 3:**
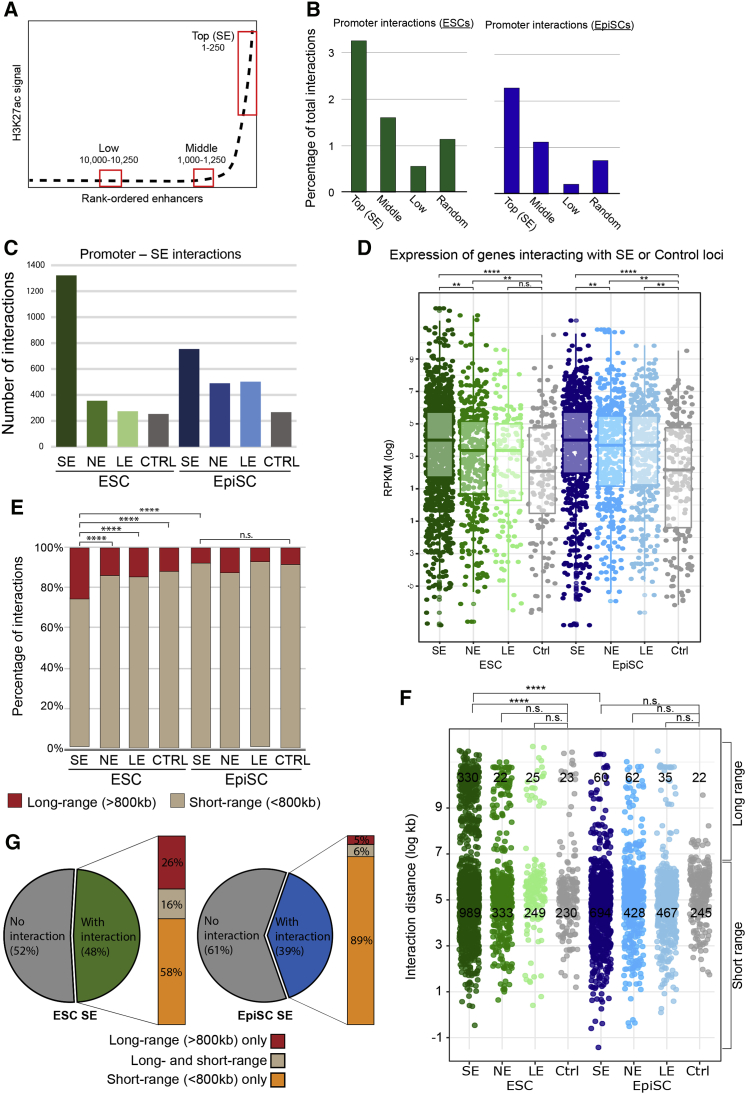
Long-Range Promoter-SE Interactions Are Prevalent in ESCs (A) After ROSE analysis, we selected three enhancer categories: the top 250 H3K27ac-ranked regions (comprising a subset of SEs), the first 250 enhancers immediately below the SE-threshold (middle), and 250 enhancers with low levels of H3K27ac. Red boxes highlight the selected enhancers within each category. (B) Proportion of PCHi-C interactions detected for the three enhancer categories and control regions in ESCs (left) and in EpiSCs (right). Control regions were randomly selected and size-matched to SEs. (C) Number of interactions after concatenation of the fragments spanning an individual SE, normal enhancer (NE), low enhancer (LE), or randomly selected control regions (CTRL) in ESCs and EpiSCs. (D) Expression (log2 reads per kilobase per million mapped reads [RPKM]) of genes interacting with the three classes of enhancers and control regions in ESCs and EpiSCs. ^∗∗∗∗^p < 0.0001; ^∗∗^p < 0.01 (Mann Whitney *U* test). (E) ESCs have a high proportion (25%) of promoter-SE interactions spanning >800 kb (red) when compared to interactions in EpiSCs or to size matched NEs and control regions (CTRL; Benjamini-Hochberg adjusted chi-square p < 0.0001). (F) A significant proportion of LRIs between promoters and SEs in ESCs (green, 25%) compared to EpiSCs (blue, 7%) or to size- and distance-match random control regions (light green, 13%; Benjamini-Hochberg adjusted p < 0.0001). NE and LE are not significantly different from controls. See Table S2 for a list of LRIs. (G) Classification of concatenated SE fragments according to the genomic distance of their target promoters. In ESCs, 58% of interacting SE regions only engage in short-range (<800 kb), 26% in long-range only (>800 kb), and 16% in both short- and long-range promoter interactions. In EpiSCs, most interacting SEs (89%) engage in short-range interactions only. Note that the proportion of interactive SE regions is likely to be underestimated due to the stringent statistical analysis applied to the concatenated regions, and also due to the inability to detect interactions when the baited promoter and SE are within neighboring HindIII fragments. See also Table S2.

To investigate whether the rewiring of interactions between pluripotent states was a consequence of altered SE status, we defined SEs in EpiSCs by running H3K27ac ChIP-seq data (Factor et al., 2014) through the SE-calling ranking of super-enhancer (ROSE) pipeline (Whyte et al., 2013). H3K27ac levels are one of the most informative features to identify SEs (Lovén et al., 2013, Khan and Zhang, 2016) and have been used to operationally define SEs in a range of tissues and species (Whyte et al., 2013, Chapuy et al., 2013, Ding et al., 2015, Pérez-Rico et al., 2017, Cao et al., 2017). This approach identified 896 SEs in EpiSCs (EpiSC-SE). To generate a comparable list in ESCs, we re-called SEs using the same pipeline, resulting in 927 SEs (ESC-SE), which incorporated the vast majority (85%) of the 231 ESCs SEs considered thus far. As expected, the number of ROSE-called SEs is higher than the original 231 ESCs SEs, as MED1 occupancy was initially used as additional criteria (Whyte et al., 2013). Approximately one-third of the ROSE-called SEs were the same in ESCs and EpiSCs. In both cell types, SEs were highly interactive when compared to “regular” enhancers or control regions that were randomly selected to size match each individual SE (Figures 3A and 3B).

To facilitate the comparison of promoter-SE interactions between ESCs and EpiSCs, we modified the CHiCAGO pipeline to concatenate all the individual HindIII fragments that overlapped a single ROSE-called SE and mapped significant interactions between promoters and each SE region. We also selected additional categories of regions with medium (normal enhancers [NEs]), low (low enhancers [LEs]) or undetectable (control [CTRL]) levels of H3K27ac and extended their sizes to match those of SE regions. As before, there were many more significant interactions with the concatenated SE regions as compared to the size-matched regular enhancers or control regions, thereby demonstrating that SEs are contacted by a particularly high number of promoter interactions (Figure 3C). The transcriptional output of genes interacting with SEs was higher than from the set of genes contacting NE, LE, or control regions (Figure 3D).

Interestingly, the proportion of long-range interactions (LRIs; defined as >800 kb) between promoters and SEs was significantly higher in ESCs than in EpiSCs. Overall, 25% of promoter-SE interactions spanned >800 kb in ESCs, which is more than for regular enhancers and control regions (p < 0.0001) and also when compared to SEs in EpiSCs (p < 0.0001; Figures 3E and 3F). Indeed, while 58% of the ESC SEs were involved in short-range interactions, 26% of the interacting SEs were engaged exclusively in LRIs and 16% in both (Figure 3G). Importantly, when considering all significant promoter-genome interactions, there was no difference in the interaction distance when comparing between ESCs and EpiSCs (Figure S2B). In addition, interaction distances between promoters and non-SE regions, such as the Polycomb-mediated *Hox* network (Schoenfelder et al., 2015b, Joshi et al., 2015), were similar in ESCs and EpiSCs (Figure S2C), suggesting that the enrichment for LRI is a specific feature of ESC SEs. Furthermore, the CHiCAGO scores for long-range and short-range SE interactions were comparable (Figure S2D). Taken together, our results reveal a prevalence for SEs to establish long-range promoter interactions in ESCs, but not EpiSCs.

### Long-Range Regulatory Contacts in ESCs Are Enriched for NANOG Occupancy

**Figure 4:**
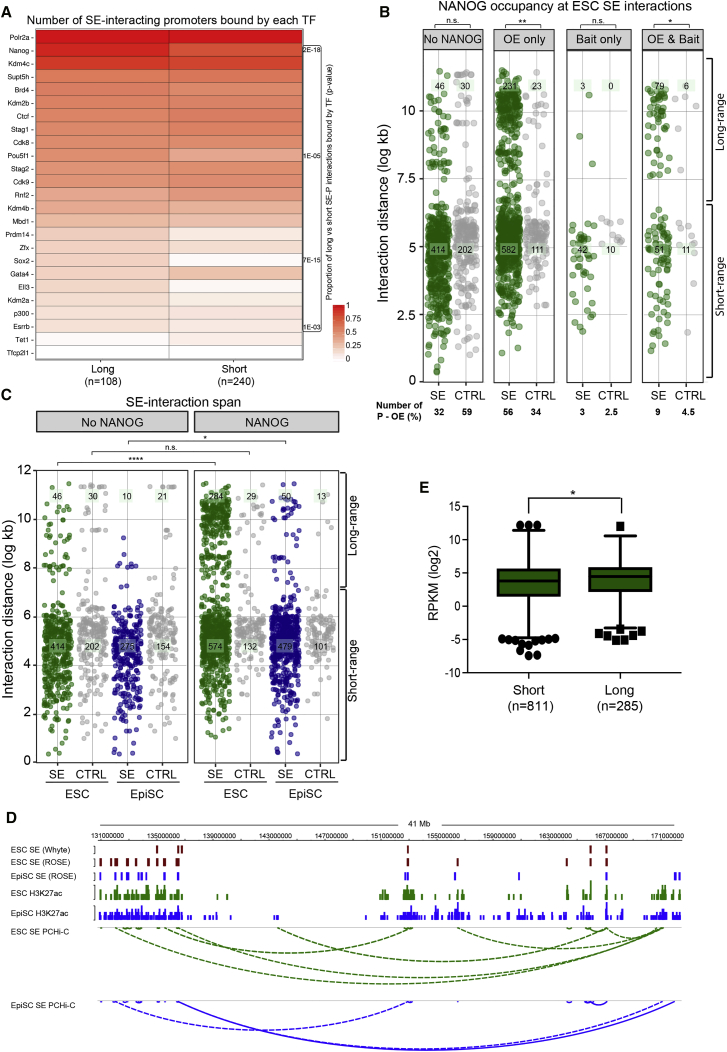
Long-Range Promoter-SE Interactions Are Enriched for NANOG Binding (A) CODEX database was integrated to screen for the binding of transcription factors (TFs) at the promoter of genes involved in long- and short-range SE interactions in ESCs. The heatmap shows the top 25 TFs bound at promoters engaged in LRIs with SEs, normalized by column. The side panel shows the Benjamini-Hochberg adjusted chi-square p values for the proportion of long- versus short-range interactions bound/not bound by NANOG, POU5F1, SOX2, and ESRRB. (B) Either end of the promoter-SE/CTRL contacts was classified according to the binding of NANOG. The majority (68%) of promoter-SE interactions in ESCs were bound by NANOG, particularly at the SE end (56%). The percentage of interactions within each category is shown. OE, other end of interaction (either SE or matched control). ^∗∗^p < 0.01; ^∗^p < 0.05 (Mann Whitney *U* test). (C) LRIs were significantly enriched for NANOG occupancy when compared to short-range (<800 kb) interactions in ESCs (green dots, Benjamini-Hochberg adjusted chi-square p = 2E-18; Figure S3A). This enrichment was moderately significant in EpiSCs (blue dots, Benjamini-Hochberg adjusted chi-square p = 8E-03) and not significant at control regions (gray dots). (D) Genome browser illustrating an example of LRIs between promoters and SEs in ESCs (green dashed arcs) and in EpiSCs (blue dashed arcs). (E) Gene expression levels (log2 RPKM) of genes engaged in long- and short-range SE interactions in ESCs (Mann-Whitney *U* test; p = 0.01). See also Figure S3 and Tables S2 and S3.

As a first step toward understanding the determinants of LRIs, we examined the CODEX ChIP-seq database (Sánchez-Castillo et al., 2015) for transcription factors that are bound at gene promoters engaged in long-range versus short-range SE interactions in ESCs. Interestingly, the binding of several pluripotency factors, including NANOG, OCT4, SOX2, and KLF4, were significantly enriched at long-range SE interactions compared to short-range ones (Figures 4A and S3A). In particular, the pluripotency factor NANOG was the second most prevalent factor at promoters that interact with SEs (present at 12% of all promoters contacting ESC SEs) and with a skew toward those promoters connected by LRIs (Figure 4A). Moreover, when considering both ends of an interaction, NANOG binds the majority (68%) of sites that overlap with promoter-SE contacts (Figure 4B), with a strong preference for binding at the SE end of the interaction only (56%). This extends the observations that NANOG is a core protein that occupies many SEs in ESCs (Whyte et al., 2013) and that large regions harboring clusters of NANOG preferentially interact (de Wit et al., 2013).

Examining this in further detail, we found that long-range promoter-SE interactions were bound by NANOG; 284 are NANOG-bound versus 46 without NANOG, whereas in contrast, the short-range SE-interactions did not show this enrichment (574 bound versus 414 not bound; Figures 4B–4D and S3A). In contrast, there was no significant difference in NANOG occupancy at interactions between promoters and randomly selected control regions that were matched for fragment size and distance from the bait (p = 0.39; Figures 4C and S3A). Although there were much fewer long-range SE interactions in EpiSCs than ESCs (total 60), we detected a modest but significant association with NANOG occupancy at long-range SE interactions compared to short-range ones (p = 0.008; Figures 4C and 4D). We found no enrichment for other genomic features like Cohesin or POLII occupancy (Figure S3B). Finally, the expression of genes involved in long-range SE interactions was slightly higher than that of genes engaged in short-range SE interactions (p = 0.01, Mann-Whitney test; Figure 4E). Taken together, long-range SE contacts in ESCs are associated with the presence of pluripotency transcription factors, in particular NANOG, and have increased expression levels compared to genes contacting SEs over a shorter distance.

### Long-Range SE Contacts Are Depleted in *Nanog*-Deficient ESCs

**Figure 5:**
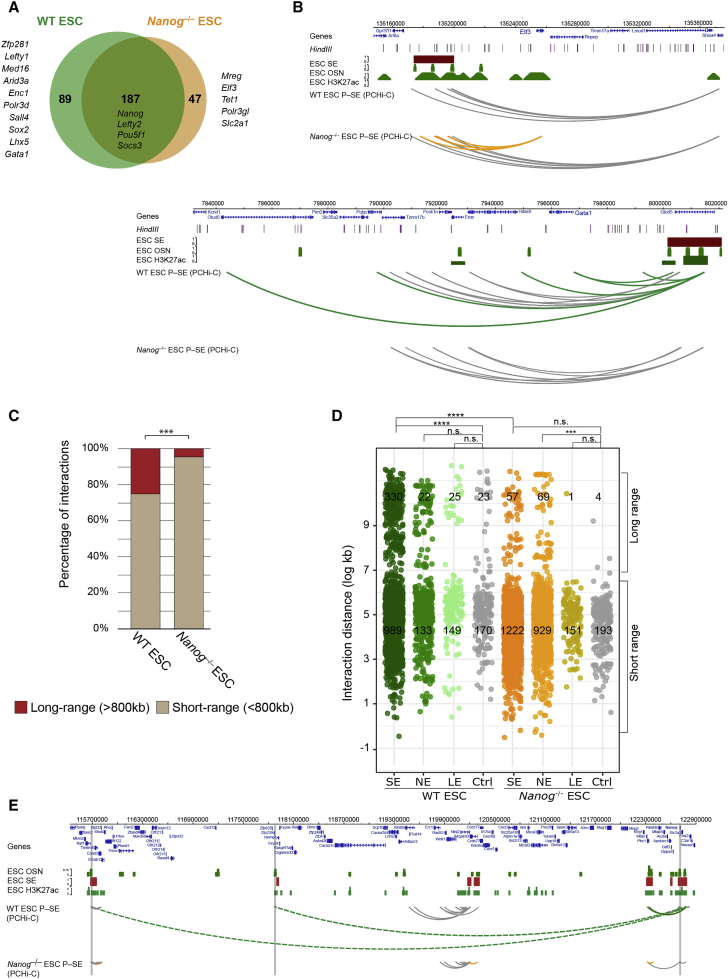
Long-Range Promoter-SE Interactions Are Depleted in *Nanog*-Deficient ESCs (A) Comparison of the SE-interactome between WT and *Nanog*^−/−^ ESCs. The Venn diagram shows the overlap of genes that significantly interact with the 231 SEs (Whyte et al., 2013) in wild-type (WT) ESCs and *Nanog*^−/−^ ESCs. A similar overlap was detected for ROSE-called WT SEs (not shown). (B) Examples of significant promoter-SE interactions in *Nanog*^−/−^ ESCs (top, *Elf3*) and in WT ESCs (bottom, *Gata1*). Significant interactions common to WT and *Nanog*^−/−^ ESCs are shown as gray arcs, while orange or green arcs represent interactions detected only in the knockout or in the WT ESCs. (C) The proportion of long-range promoter-SE interactions was significantly reduced in *Nanog*^−/−^ ESCs compared to WT ESCs (57 versus 330 LRIs, respectively; Benjamini-Hochberg adjusted chi-square p < 0.0001). (D) The proportion of long-range versus short-range promoter-SE interactions in WT ESCs is significantly higher compared to *Nanog*^−/−^ ESCs (Benjamini-Hochberg adjusted chi-square p < 1E-05). No significance in the proportion of long- versus short-range was detected between promoter-SE and promoter-CTRL in *Nanog*^−/−^ ESCs. (E) Promoter-SE interactions at a large region encompassing the *Nanog* locus. Grey arcs show promoter-SE interactions common to both WT and *Nanog*^−/−^ ESCs, and orange and green arcs represent interactions detected in each, respectively. Dashed arcs indicate LRIs. Vertical gray lines denote SE interactions at sites bound by the pluripotency transcription factors OCT4, SOX2, and NANOG (OSN). See also Figure S3 and Tables S1 and S3.

Although the occupancy of core transcription factors is a defining feature of SEs, it is not known whether the transcription factors themselves have a role in establishing SE contacts. To investigate a potential role for NANOG in coordinating the SE interactome, we generated PCHi-C libraries from *Nanog*^−/−^ ESCs. Although depleted in a core transcription factor, *Nanog*-deficient cells retain key properties of ESCs, including the ability to self-renew and to undergo multi-lineage differentiation (Chambers et al., 2007). We identified the gene promoters that interacted with SEs in *Nanog*^−/−^ ESCs and compared this list to those that we assigned in wild-type (WT) ESCs. Overall, ∼80% (187) of the genes interacting with SEs in *Nanog*^−/−^ ESCs are common to WT ESCs, demonstrating that NANOG is not essential to maintain most promoter-SE interactions in ESCs. Notably, we did identify a set of gene promoters that interacted with SEs only in *Nanog*^−/−^ ESCs, such as *Elf3* and *Tet1* (47; 20%), and another set that interacted with SEs only in WT ESCs, including *Zfp281* and *Lefty1* (87; Figures 5A and 5B).

Interestingly, some of the changes in SE interactions upon *Nanog* deletion resembled the differences observed between ESCs and EpiSCs. For example, 24% of the newly interacting promoters in *Nanog*^−/−^ ESCs were also present in EpiSCs (Figure S3C). Moreover, LRIs were particularly susceptible to the deletion of *Nanog*, as they were 6-fold reduced in *Nanog*^−/−^ ESCs compared to WT ESCs (4% of all promoter-SE interactions span ≥800 kb, compared to the 25% in WT ESCs, p < 0.0001; Figures 5C–5E). Deleting *Nanog* in ESCs, therefore, triggers an interaction rewiring that partially recapitulates the differences between ESCs and EpiSCs. These findings lead us to propose that the presence of NANOG contributes to the stability of a subset of ESC-specific SE contacts, and particularly those interactions that span large distances.

## Discussion

In recent years, a subset of regulatory elements termed SEs have gained attention across a wide range of research fields (Hnisz et al., 2013, Whyte et al., 2013, Parker et al., 2013, Lovén et al., 2013). The interest in SEs stems from their potential to act as responsive, regulatory hubs that can control cell identity. Equally, questions have been raised as to whether SEs should be considered as separate entities from clusters of canonical enhancers or locus control regions (Moorthy et al., 2017, Bojcsuk et al., 2017, Hay et al., 2016, Pott and Lieb, 2015), and thus, it is fundamental to investigate the properties of SEs.

In this study, we generated high-resolution, global maps of promoter interactions in two distinct pluripotent cell types, ESCs and EpiSCs. We found that SEs form complex spatial networks in which individual SEs have the potential to regulate several genes. This observation expands on the one promoter to one SE assignments that have been proposed using Hi-C data and computational predictions (Whyte et al., 2013, Ing-Simmons et al., 2015). Whether this multi-modular form of gene regulation ensures robustness of transcriptional control or opportunities for co-regulation remains to be tested experimentally. The latter hypothesis is supported by a previous study where deleting an individual SE triggered the dysregulation of several nearby genes (Moorthy et al., 2017). Extending these observations to additional SEs, particularly in association with our promoter-SE interactome, will provide an exciting line of future studies.

Genes assigned to SEs have been proposed to confer unique cellular identities (Parker et al., 2013, Hnisz et al., 2013). By comparing the SE interactome of ESCs and EpiSCs, we detected the gain and loss of significant SE interactions at a subset of potentially important loci. For example, we detected a significant interaction between the *Klf4* promoter and a SE in ESCs only and between *Klf13* and a SE only in EpiSCs. Thus, the rewiring of SE interactions at genes encoding transcription factors could underlie the regulatory signals required for pluripotent state transitions. This is further supported by the differential enrichment for the binding of distinct sets of transcription factors at genes interacting with SEs in ESCs and EpiSCs, which might stabilize active SE interactions in ESCs while promoting the “priming” of new SE interactions upon transition to EpiSCs. However, we also found many genes interacting with SEs in both cell types that are not currently associated with pluripotency. For example, we uncovered a subset of genes engaged in SE interactions in ESCs with functions related to the synthesis of acetyl-coenzyme A (acetyl-CoA). The glycolytic production of acetyl-CoA promotes histone acetylation and helps to maintain the “open” chromatin organization of ESCs (Moussaieff et al., 2015). By connecting genes to highly active regulatory elements, our PCHi-C resource could be used to identify additional and previously unexplored mediators of pluripotent states.

Although the occupancy of core transcription factors is a hallmark of SEs, whether such factors contribute to SE function has not been investigated previously. We found that the deletion of *Nanog*, which is a SE-associated transcription factor in ESCs, had a surprisingly modest impact on the majority of promoter-SE contacts. However, we did observe that particular subsets of SEs were disrupted in several important ways. First, we detected a reduction in the span of promoter-SE interactions, such that significant LRIs were less frequent in *Nanog*-deficient ESCs. Second, interaction rewiring resulted in a different set of promoters in contact with SEs. We are unable to distinguish between whether the interaction differences were driven predominantly by the loss of SE activity in *Nanog*-deficient ESCs or by the loss of the interaction itself. We also cannot rule out that some of these changes could be due to the increased prevalence of partially differentiated cells within the *Nanog*-deficient cultures. Nevertheless, it is interesting to note that both of these genomic changes resembled the SE interactome in EpiSCs. Given that NANOG levels are lower in EpiSCs than ESCs, differences between the pluripotent states seem to be accompanied by a reorganization of the network of promoters contacting regulatory regions. In particular, this involves the decommissioning of regulatory regions associated with promoters that are involved in pluripotency/stem cell maintenance (like *Zfp281* or *Sall4*) and the rewiring to promoters that are involved in chromatin organization and differentiation priming (such as *Tet1*, *Elf3*, and *Slc2a1*).

Another interesting feature that emerged from our analysis was the prevalence of LRIs between promoters and SEs in ESCs, with 25% of such interactions spanning 800 kb to 90 Mb. Notably, these LRIs were rarely detected in EpiSCs. This finding suggests that the ESC genome has a permissive organization, which enables a more highly folded spatial arrangement with DNA loops that can span greater distances. Interestingly, previous studies reported that ESCs form long-range promoter-promoter interactions, mainly involving the *Hox* clusters and other Polycomb-associated sites (Denholtz et al., 2013, Joshi et al., 2015, Schoenfelder et al., 2015b). In addition, a recently developed method called genome architecture mapping identified chromatin contacts from thin nuclear sections and found that TADs containing SEs are highly interactive in ESCs and their contacts can span up to 116 Mb (Beagrie et al., 2017). Together with our findings, this leads to a model in which ESCs are particularly permissive to establishing physical contacts with very distant sites, both at transcriptionally repressed promoters and at highly active promoter-SE regions. Importantly, very few significant long-range SE contacts were detected in EpiSCs or in *Nanog*^−/−^ ESCs, suggesting that it might be a feature resultant from the distinct nuclear organization of WT ESCs. Indeed, we and others have previously shown that ESCs have a more open and dynamic chromatin architecture than EpiSCs or *Nanog*^−/−^ ESCs (Meshorer and Misteli, 2006, Fussner et al., 2011, Efroni et al., 2008, Novo et al., 2016). One interesting exception to this is that LRIs at the *Hox* network seem to be unaffected by the global changes in chromatin organization, as these interactions remained in EpiSCs. Further experiments are needed to define how Polycomb-group proteins are able to control the large spatial *Hox* networks within this environment. As long-range promoter-SE interactions also seem to be largely associated with NANOG, we hypothesize that the increased chromatin compaction that is triggered by reduced NANOG levels in EpiSCs or deletion of *Nanog* in mutant ESCs could restrict the connectivity between SEs and their distal target promoters. Indeed, although there were few long-range contacts involving control regions, this number was further reduced in *Nanog*-depleted cells, which points toward a global constriction in interaction distances. Alongside these global effects, it is possible that NANOG also has a direct role in stabilizing long-range SE interactions in ESCs, and future work could examine this prediction through acute gain and loss of NANOG binding.

A regulatory network where several gene promoters are in close proximity to strong regulatory elements like SEs confers the potential for a rapid and dynamic response to altered stimuli. For example, variations in the recruitment of transcription factors or signaling pathway members to SEs could quickly impact the regulation of the genes in contact with the SEs. Interestingly, the joint engagement of multiple promoters and enhancers in “chromatin hubs” has been observed in other contexts, and it is possible that SEs could be involved in a similar mode of gene regulation (Tolhuis et al., 2002, Patrinos et al., 2004, Jiang et al., 2016). We did not find a correlation between global transcription changes and SE interactions, suggesting that the spatial association between a promoter and a SE is not predictive of the cell-type-specific transcriptional status of that gene and that additional features (e.g., other regulatory inputs, binding of transcription factors, chromatin accessibility) mediate the differential gene expression changes. Contacts between multiple active regions are common in individual cells (Beagrie et al., 2017), and single-cell approaches will be particularly valuable for interrogating the potential of individual SEs for multiple gene regulation.

Taken together, our work provides an annotated view of interactions between putative regulatory elements, including SEs, and their target promoters across multiple pluripotent cell types. These results lead us to conclude that SEs form spatial networks in pluripotent cells that are partly dependent on core transcription factor occupancy. The data can be used to better define the gene regulatory architecture in pluripotency and understand the transcriptional variability that is observed upon perturbation of regulatory elements. This work will also be informative for understanding gene regulation in other systems including adult stem cells (Adam et al., 2015) and alternative developmental models, and it has valuable applications for cell therapies that are currently focused on targeting SEs as drivers of disease (Hnisz et al., 2013).

## Experimental Procedures

### PCHi-C Quantification and Statistical Analysis

Raw sequencing reads were processed using Hi-C User Pipeline (HiCUP) (Wingett et al., 2015). Interactions were called using CHiCAGO (Cairns et al., 2016), and the resulting p values were adjusted with a weighted false discovery control procedure. Interactions were called at the level of individual HindIII fragments based on two biological replicates for each cell type that were normalized and combined. Interactions with a CHiCAGO score ≥5 were classified as high-confidence interactions. Additionally, interactions with scores ≥4 were included in the analysis if they scored >5 in one biological replicate and ≤5 in both replicates of the other cell types. Table S1 lists significant interactions.

### SE Calling

H3K27ac ChIP-seq data for ESCs (Creyghton et al., 2010) and EpiSCs (Factor et al., 2014) were mapped to GRCm38/mm10. SEs were identified by ROSE (https://bitbucket.org/young_computation/rose) as described previously (Lovén et al., 2013, Whyte et al., 2013). Briefly, after model-based analysis of ChIP-seq (MACS) peaks identification, peaks within 12.5 kb distance of each other were stitched with a TSS exclusion zone size of 2 kb. The signal of stitched enhancers was determined by the total normalized number of reads minus the number of normalized reads in the input. Stitched and normalized peaks were then ranked by H3K27ac density and those higher than the inflection point on the density curve were defined as SEs (genome locations in Table S2).

### SE Interaction Calling

We improved the power of promoter-SE interaction calling by “stitching” the fragments together. Only *cis*-chromosomal interactions were considered. For each promoter-SE interaction test, we stitched together all HindIII fragments overlapping the appropriate SE, except for the bait and its adjacent fragments. We then assigned an observed and expected read count to each promoter-SE pair as follows. The observed count is the total number of reads between the baited fragment and any of the HindIII fragments overlapping the SE. Similarly, the expected count for the composite interaction is the sum of the expected Brownian counts retrieved for each bait-HindIII-fragment pair. Finally, we tested if the observed count was greater than expected by performing a one-tailed hypothesis test - the null distribution was Negative Binomial, with mean equal to the expected count and with dispersion parameter retrieved from the original CHiCAGO analysis, thereby obtaining a p value. The p values were weighted and transformed into scores using the default methods of CHiCAGO (Cairns et al., 2016), and scores ≥5 were classified as high-confidence interactions. Randomized control regions were defined by shuffling SEs within each chromosome until no two control regions overlapped. NE and LE regions were defined by H3K27ac signals. Each region was assigned randomly to an SE, and the regions were size extended from the midpoint of the fragment until their size matched the assigned SE. Extended regions that shared a fragment with an SE were discarded and replaced with an alternative size-matched region. A list of the interactions can be found in Table S2.

### LRI

The midpoints of each interacting genomic feature were subtracted to define the distances of individual interactions: distance = |(a + b)/2 − (c + d)/2|, where (a,b) and (c,d) are the (start,end) coordinates of each interacting genomic feature. 25% of SE-interactions in ESCs span >800 kb, and this value was used as the threshold to define LRIs. To calculate the LRI enrichment, we compared the proportion of SE interactions spanning ≥800 kb in each cell type. The proportion of LRI versus short-range (<800 kb) SE interactions was calculated and represented as percentages of all SE interactions for a cell type. Any proportion differences of LRI versus short-range SE-interactions between cell types was tested using the chi-square test of independence.

## Data and Software Availability

The accession number for the raw and processed sequencing data reported in this paper is GEO: GSE103053.

## Supplemental Information

Document S1. Supplemental Experimental Procedures and Figures S1–S3

Table S1. Gene Promoter Assignment to SEs by Mapped Physical Interaction and Significant PCHi-C Interactions for 231 ESC SEs, Related to Figures 1, 2, and 5 Lists of all significant interactions with 231 ESC SEs (Whyte et al. 2013) in ESCs, EpiSCs and *Nanog*−/− ESCs.

Table S2. ROSE SE Coordinates and Significant PCHi-C Interactions, Related to Figures 2, 3, 4, and 5 Genomic locations of ROSE-called SEs in ESCs and EpiSCs. Lists of all significant interactions with the ROSE-called SEs in ESCs and EpiSCs.

Table S3. ChIP Datasets Used in This Study, Related to Figures 4 and 5 A list of the datasets used for ChIP analysis and their accession codes.

Document S2. Article plus Supplemental Information
