# Supplementary material for: Long-Range Enhancer Interactions Are Prevalent in Mouse Embryonic Stem Cells and Are Reorganized upon Pluripotent State Transition
Source: Cell Rep. 2018 Mar 6;22(10):2615–27. doi: 10.1016/j.celrep.2018.02.040 (PMC5863031; doi:10.1016/j.celrep.2018.02.040)
Supplement: Document S1. Supplemental Experimental Procedures and Figures S1–S3 [file mmc1.pdf]

Cell Reports, Volume *22*

Supplemental Information

Long-Range Enhancer Interactions Are Prevalent in Mouse Embryonic Stem Cells and Are Reorganized upon Pluripotent State Transition

Clara Lopes Novo, Biola-Maria Javierre, Jonathan Cairns, Anne Segonds-Pichon, Steven W. Wingett, Paula Freire-Pritchett, Mayra Furlan-Magaril, Stefan Schoenfelder, Peter Fraser, and Peter J. Rugg-Gunn

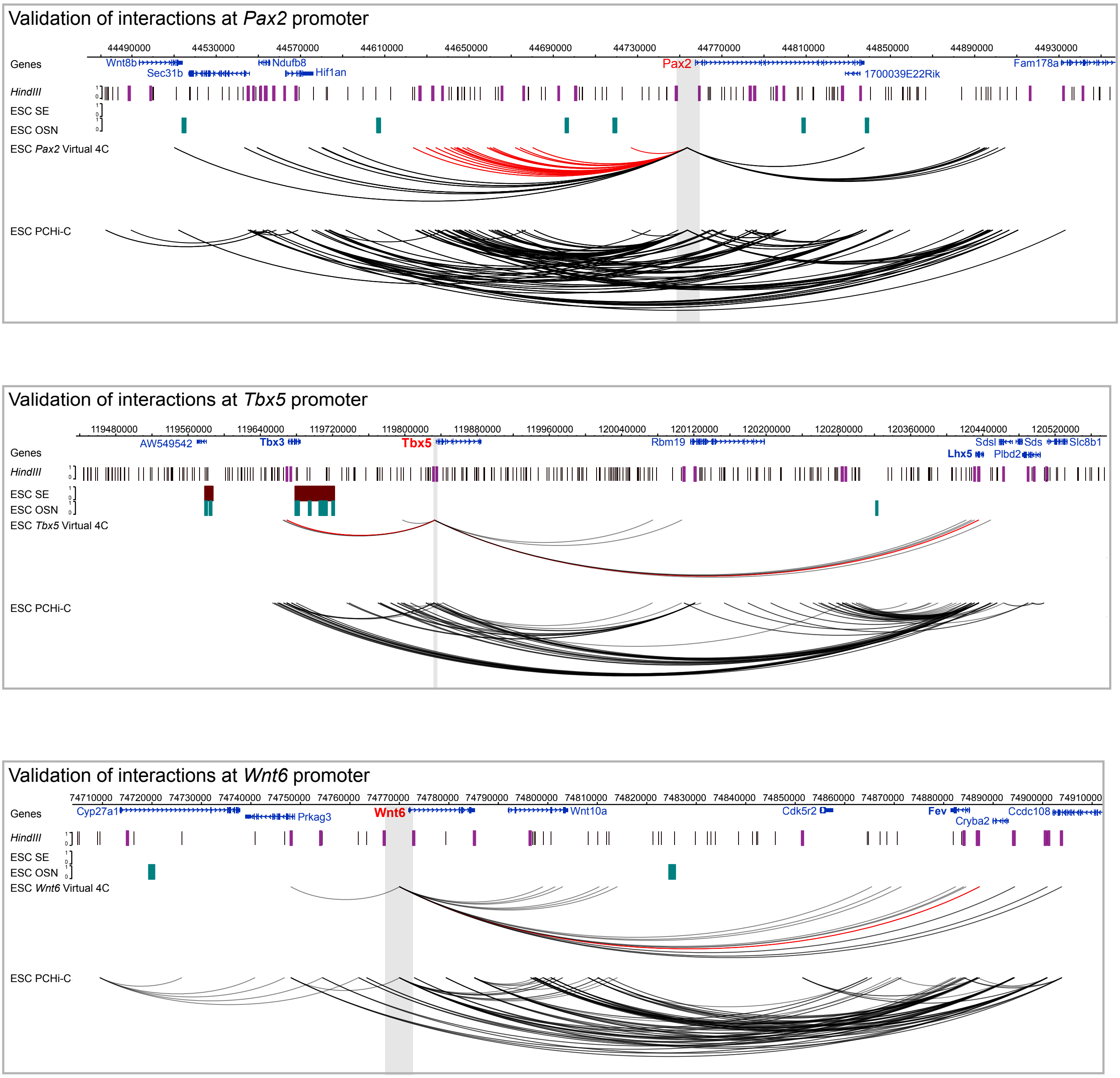

Figure S1, related to Figure 1 - Validation of PCHi-C libraries

By performing a virtual 4C analysis, previously described ESC promoter interactions were also found in our libraries.For example, the *Pax2* promoter interacts with a nearby putative enhancer region (top pannel, (Schoenfelder, S. et al. 2015*a*). We also detected the previously described *Tbx5* interactions with *Lhx5* and with *Tbx3* (middle) as well as the *Wnt6* interaction with *Fev* (bottom, Schoenfelder, S. et al 2015*b*). Validated interactions, red arcs. All other interactions, black arcs. Baited *HindIII* fragments, purple.

a Schoenfelder, S., R. Sugar, A. Dimond, et al. 2015. 'Polycomb repressive complex PRC1 spatially constrains the mouse embryonic stem cell genome', Nat Genet, 47: 1179-86.

b Schoenfelder, S., M. Furlan-Magaril, et al. 2015. 'The pluripotent regulatory circuitry connecting promoters to their long-range interacting elements', Genome Res, 25: 582-97.

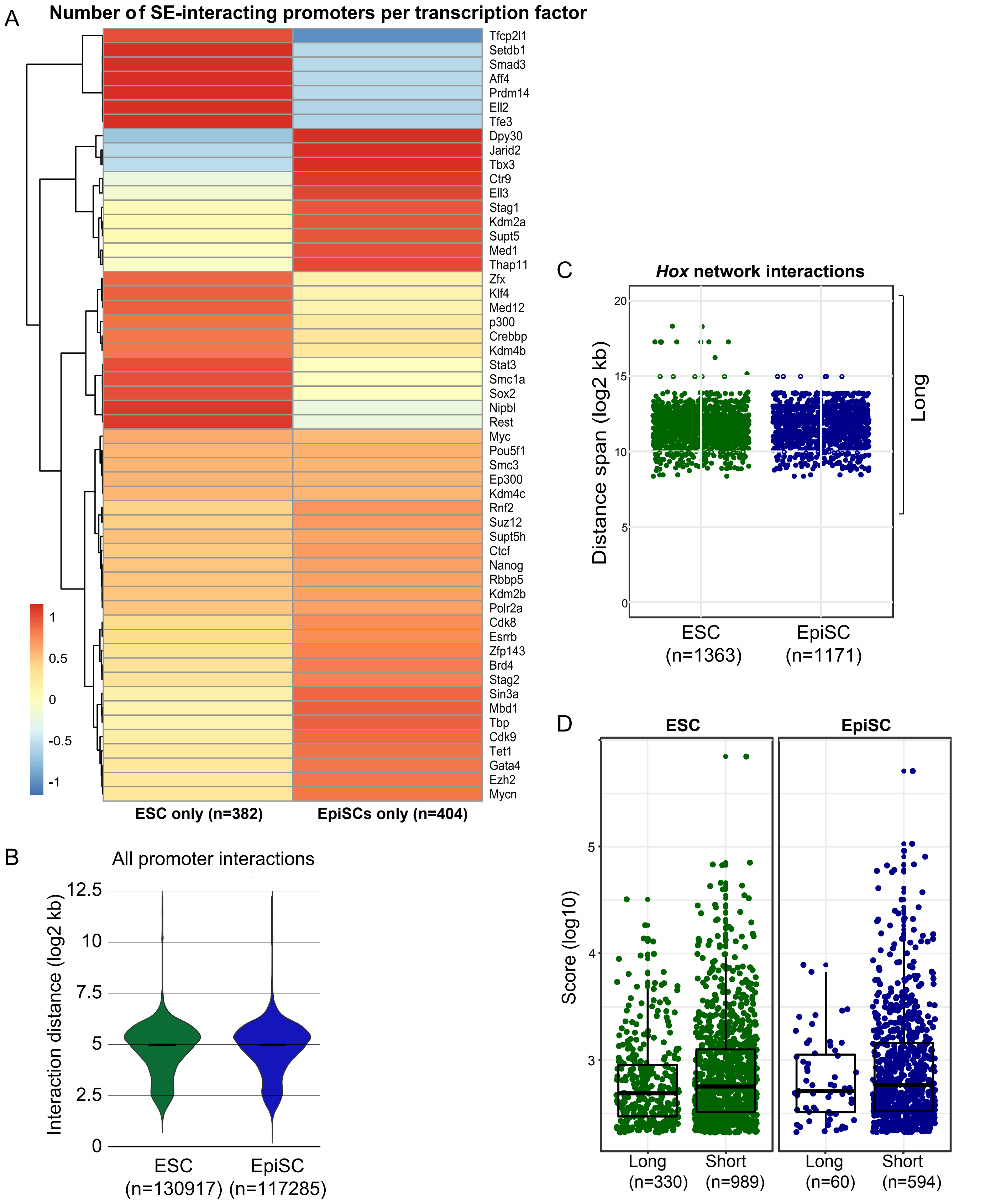

Figure S2, related to Figure 2 - Characterization of SE interactome

a) The number of SE-interacting promoters bound by each transcription factor in the CODEX ChIPseq database was calculated. The heatmap shows these values, mean centred and scaled by row, for ESC-only and EpiSC-only sets.**b)** Violin plot of the genomic distances (log2 kb) of all significant interactions in ESCs and EpiSCs.

c) Genomic distances (log2 kb) of significant interactions within the *Hox* network in both pluripotent states.

d) Dotplot of CHiCAGO scores of long- and short-range interactions between SE and promoters, in both cell-lines.

A

	NANOG (%)		SOX2 (%)		OCT4 (%)		CTCF (%)		KLF4 (%)	
	Long	Short	Long	Short	Long	Short	Long	Short	Long	Short
**ESC SE**	86 **(2E-18)**	58	82 **(7E-15)**	56	98 **(1E-05)**	89	98 (4E-02)	94	37 **(1E-06)**	22
**ESC CTRL**	49 (3E-01)	40	44 (6E-02)	28	69 (4E-01)	61	76 (2E-02)	90	10 (5E-01)	15

B

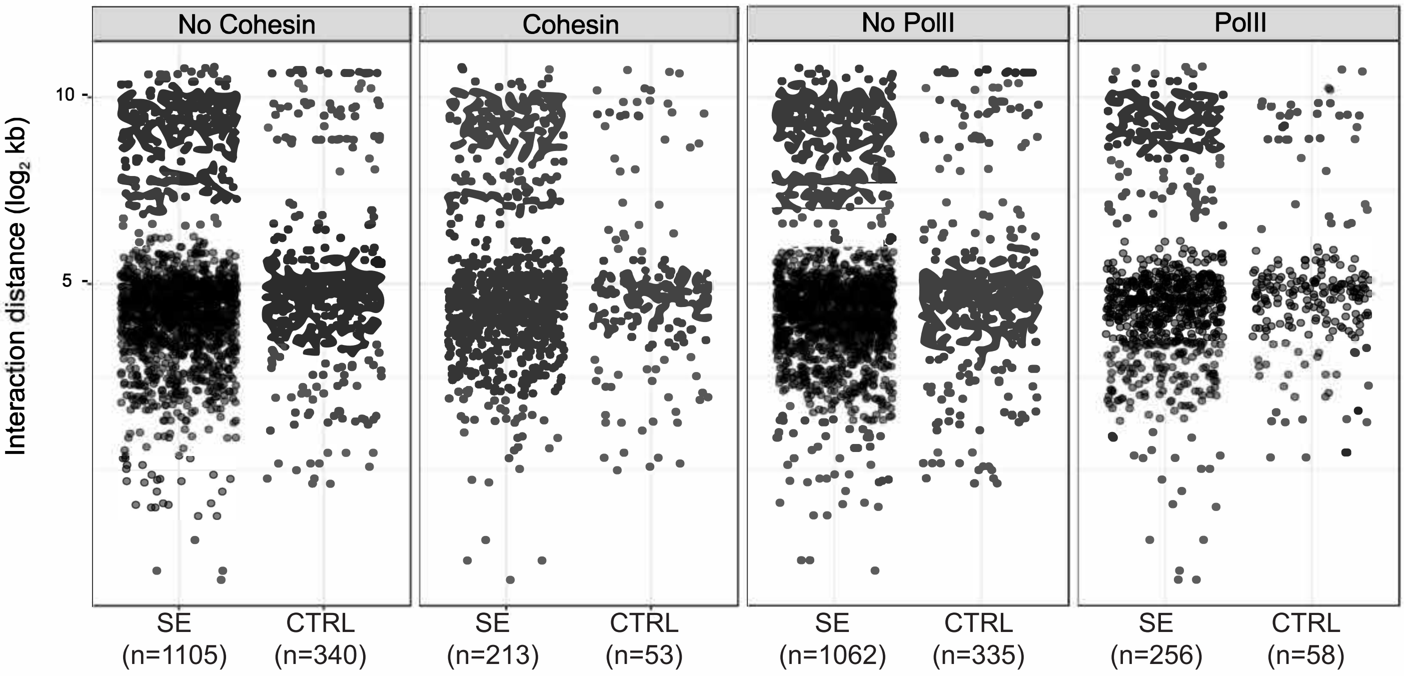

C

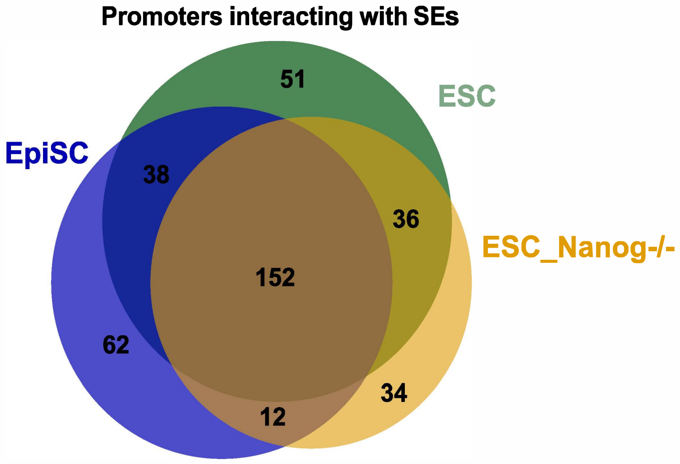

Figure S3, related to Figure 4 - Screening for different genomic features at SE-P in all cell-types analysed.

a) Percentage of long-range (>800kb) or short-range (<800kb) interactions bound by different proteins inESCs for SEs and for control regions (CTRL). In brackets, the Benjamini-Hochberg adjusted Chi-squared p-values for the pairwise comparison between the proportion of bound/not bound in long-range versus short-range interactions are shown. Significant differences are highlighted in bold.

b) Each SE or Control - P interaction was annotated for the binding of Cohesin or POUi and plotted according to the genomic distance.

c) Venn-diagram with the promoters interacting with SE (Whyte et al 2013) in the three pluripotent cell-lines analysed.

Supplemental Experimental Procedures

Cell culture
E14Tg2a (129P2/OlaHsd (Hooper et al., 1987) and RCNβH-B(t) (E14Tg2a-derived Nanog$^{-/-}$, passages 20-30) (Chambers et al., 2007) ESCs were cultured on gelatin-coated surfaces in standard ESC media (DMEM supplemented with 15% FBS, 1mM sodium pyruvate, 0.1mM 2-mercaptoethanol, 0.1mM non-essential amino acids, 2mM glutamax and 1000 U/ml LIF), and 25µg/ml hygromycin was added during RCNβH-B(t) ESC expansion to select for undifferentiated Nanog$^{-/-}$ cells. Embryo-derived 129S2 (Brons et al., 2007) EpiSC were cultured on fibronectin (10µg/ml) in N2B27 media supplemented with 20ng/ml Activin A and 12ng/ml bFGF.

Hi-C and PCHi-C preparation
Promoter Capture Hi-C was carried out with SureSelect target enrichment, using a custom-designed biotinylated RNA bait library for mouse promoters and custom paired-end blockers according to the manufacturer's instructions (Agilent Technologies) (Schoenfelder et al., 2015a) and following the in-nucleus Hi-C protocol detailed in (Nagano et al., 2015). Briefly, HindIII-digested chromatin was end-filled with biotinylated d-ATP and ligated in preserved nuclei. After de-crosslinking, DNA was purified and sheared to an average size of 400bp (Covaris). Sheared ends were repaired and adenine-tailed prior to immobilization of the biotinylated ligation fragments using MyOne Streptavidin C1 DynaBeads (Invitrogen). Promoter capture Hi-C was performed with 500ng of Hi-C library using the custom-made RNA bait library mentioned above. After paired-end adapter ligation, libraries were amplified by PCR for four cycles.

ChIP-seq analysis and data processing
ChIP-seq in EpiSCs was performed according to (Schoenfelder et al., 2015b), with 200 µg of chromatin and 5 µg of antibody for each IP (NANOG, ab80892 Abcam; OCT4 sc-5279, Santa Cruz and SOX2, ab97959 Abcam). Libraries were prepared using the NEBNext DNA Library Preparation kit and sequenced on an Illumina HiSeq2500.

The publicly available ChIP-seq data sets used are listed in Supplementary Table 2. Raw ChIP-seq data was processed by re-alignment of reads to GRCm38/mm10 using Bowtie with default parameters (Langmead et al., 2009), followed by MACS peak-calling (Zhang et al., 2008) to generate a map of protein binding sites (using p-value of 1E-9). When raw data was not available, binding sites (peak location) and, when required, genome coordinates were converted to mm10 using the UCSC Genome Browser liftOver command line tool (https://genome.ucsc.edu/cgi-bin/hgLiftOver). Interactions were considered to be occupied by a protein factor if they overlapped at least one ChIP-seq peak (bedtools).

RNA-seq libraries and analysis
Total RNA was purified using an RNeasy kit (Qiagen). Indexed mRNA-Seq libraries were constructed from 500ng total RNA using the Tru-Seq RNA Library Prep Kit v2 (Illumina). Library fragment size and concentration was determined using an Aglient Bioanalyzer 2100 and KAPA Library Quantification Kit (KAPA Biosystems). Samples were sequenced on an Illumina HiSeq as 50bp or 100bp single-end libraries at the Babraham Institute Sequencing Facility.

Reads were trimmed using trim galore (www.bioinformatics.babraham.ac.uk/projects/trim_galore/) using default parameters to remove the Illumina adapter sequence. Reads were mapped to the mouse NCBI37 genome assembly using TopHat guided by the gene models from Ensembl. BAM files were imported to Seqmonk (www.bioinformatics.babraham.ac.uk/projects/seqmonk/). Read counts per transcript were calculated using the RNA-Seq quantitation pipeline using non-directional counts. Raw read counts per transcript were calculated using the RNA-seq quantitation pipeline on the Ensembl v70 gene set using non-directional counts. Differential analysis of gene expression was

performed using the default settings in DESeq2 (Love et al., 2014) without independent filtering of the results. Differentially expressed genes were called at padj < 0.05 and log2 fold change above 1.5 or below −1.5.

Statistical analysis

Proportions of long versus short-range and bound versus not bound interactions were compared using Chi-Square tests followed by Benjamini-Hochberg corrections to account for multiple comparisons.

CODEX analysis

The number of genes bound by each transcription factor in the CODEX ChIPseq database was calculated for both sets of genes interacting with SE in only ESC or EpiSC (ESC- and EpiSC-only, respectively) and for a set common to EpiSC and ESC. Only the CODEX ChIP-seq peak sites that overlapped TSSs were included in the analysis. The pheatmap package in R was used and values were scaled by row to enable the relative number of genes per transcription factor to be compared between the EpiSConly, ESConly and common datasets. The default scaling method in R was used so, for each set of 3 values, the data was mean centred and then divided by the standard deviation of the 3 values. For long- versus short-range interactions in ESC, the analysis performed was similar except for the scaling, which was performed by column using the dplyr package in R.

Supplemental References

BRONS, I. G., SMITHERS, L. E., TROTTER, M. W., RUGG-GUNN, P., SUN, B., CHUVA DE SOUSA LOPES, S. M., HOWLETT, S. K., CLARKSON, A., AHRLUND-RICHTER, L., et al. 2007. Derivation of pluripotent epiblast stem cells from mammalian embryos. Nature, 448, 191-5.

CHAMBERS, I., SILVA, J., COLBY, D., NICHOLS, J., NIJMEIJER, B., ROBERTSON, M., VRANA, J., JONES, K., GROTEWOLD, L. & SMITH, A. 2007. Nanog safeguards pluripotency and mediates germline development. Nature, 450, 1230-4.

HOOPER, M., HARDY, K., HANDYSIDE, A., HUNTER, S. & MONK, M. 1987. HPRT-deficient (Lesch-Nyhan) mouse embryos derived from germline colonization by cultured cells. Nature, 326, 292-5.

LANGMEAD, B., TRAPNELL, C., POP, M. & SALZBERG, S. L. 2009. Ultrafast and memory-efficient alignment of short DNA sequences to the human genome. Genome Biol, 10, R25.

NAGANO, T., VARNAI, C., SCHOENFELDER, S., JAVIERRE, B. M., WINGETT, S. W. & FRASER, P. 2015. Comparison of Hi-C results using in-solution versus in-nucleus ligation. Genome Biol, 16, 175.

SCHOENFELDER, S., FURLAN-MAGARIL, M., MIFSUD, B., TAVARES-CADETE, F., SUGAR, R., JAVIERRE, B. M., NAGANO, T., KATSMAN, Y., et al 2015a. The pluripotent regulatory circuitry connecting promoters to their long-range interacting elements. Genome Res, 25, 582-97.

SCHOENFELDER, S., SUGAR, R., DIMOND, A., JAVIERRE, B. M., ARMSTRONG, H., MIFSUD, B., DIMITROVA, E., MATHESON, L., TAVARES-CADETE, F., et al. 2015b. Polycomb repressive complex PRC1 spatially constrains the mouse embryonic stem cell genome. Nat Genet, 47, 1179-86.

ZHANG, Y., LIU, T., MEYER, C. A., EECKHOUTE, J., JOHNSON, D. S., BERNSTEIN, B. E., NUSBAUM, C., MYERS, R. M., et al. 2008. Model-based analysis of ChIP-Seq (MACS). Genome Biol, 9, R137.
